# Chinese Artichoke (*Stachys affinis* Bunge): The Nutritional Profile, Bioactive Profile and Food Applications—A Review

**DOI:** 10.3390/molecules29153525

**Published:** 2024-07-26

**Authors:** Rafał Wiśniewski, Joanna Harasym

**Affiliations:** Department of Biotechnology and Food Analysis, Wroclaw University of Economics and Business, 53-345 Wroclaw, Poland; joanna.harasym@ue.wroc.pl

**Keywords:** proteins, oligosaccharides, polyphenolic compounds, *Stachys* L., antioxidant activity, anticancer properties

## Abstract

*Stachys affinis* Bunge, known as Chinese artichoke, is a perennial plant originating from China, which has uprising scientific interest due to its complex and beneficial content. Chinese artichoke is rich in bioactive compounds useful for human health, including antioxidants, polyphenols, and prebiotics, and its edible tubers are high in essential nutrients and dietary fiber. Studies show its potential as a functional food ingredient in various products like rice bars, bread, and chocolate, enhancing their nutritional and sensory properties. Additionally, Chinese artichoke exhibits significant anti-inflammatory, neuroprotective, and antibacterial activities, warranting further research and utilization in the food industry. This review aims to summarize the existing knowledge of the *S. affinis* Bunge plant, focusing on its health-promoting aspects.

## 1. Introduction

Bioactive compounds are prevalent throughout the plant kingdom, offering protective benefits for plants, as well as human and animal health. These bioactive substances function as natural antioxidants, potentially preventing a variety of lifestyle-related diseases [1,2]. Globally, numerous plant species rich in bioactive substances have been extensively studied [2,3]. Significant research has focused on lesser-known species of green plants or herbs, which can be potential sources of bioactive compounds and have excellent properties for industry. Increasingly, novel herbs are being incorporated into local and global food systems [3,4]. In some of these herbs the aboveground parts like leaves and flowers can be consumed, while in others the underground parts are edible like tubers or roots [5]. Additionally, these neglected and underutilized edible herb species can enhance the livelihoods of small-scale farmers and local producers [6].

The genus *Stachys* L., comprising approximately 300 species, belongs to the Lamiaceae family and is distributed globally, with a significant presence in East Asia and Europe. Many *Stachys* species have been utilized in traditional medicine since ancient Egyptian times [3,4]. These species are typically annuals or small shrubs with simple or perennial herbs or sessile leaves. They feature terminal spike-like inflorescences, with calyx tubes that are tubular-campanulate, five- or ten-veined, and weakly or regular bilabiate with five subequal teeth. The corolla features a narrow tube and is two-lipped, with an upper lip that is either flat or hooded and typically hairy, while the lower lip is three-lobed and may be either glabrous or hairy. The nutlets are oblong to ovoid, with a rounded apex [7]. The name “*Stachys*” is derived from the Greek “*Stachys*”, meaning “spike”, and in reference to the inflorescences resembling that of common wheat. The Latin term “trifarium” means tomentose [8]. Various *Stachys* species are extensively used in traditional medicine across different countries. Herbal preparations of these species are widely used for skin inflammations, and to treat stress, genital tumors, and stomach disorders [9,10]. 

Extensive phytochemical and pharmacological research have validated the traditional uses of *Stachys* species, highlighting their antioxidant, anti-inflammatory, renoprotective, analgesic, antidepressant, and anxiolytic activities [3,4,11,12]. Over two hundred bioactive compounds have been identified, including polyphenols (such as phenolic acids, lignans, phenylethanoid glycosides, and flavone derivatives), and terpenes (such as diterpenes, triterpenes, iridoids), as well as essential oils [3,4,10,13]. Due to their therapeutic and economic potential, many *Stachys* species are cultivated for traditional medicine, food, cosmetics, and ornamental purposes [4,14,15]. Despite extensive research on general *Stachys* species, recent comprehensive reviews are lacking on *Stachys affinis* Bunge as an alternative bioactive plant for the food industry. This review’s objective is to summarize current knowledge on the agronomic requirements, nutritional value, bioactive profile, and health benefits of Chinese artichoke (*S. affinis* Bunge).

## 2. Botanical Characteristics

*Stachys affinis* (called names such as Chinese artichoke, Japanese artichoke, crosne, knotroot, or artichoke betony) is a perennial herbaceous plant of the family Lamiaceae, originating from China [16]. Scientific classification: Genus—*Stachys*, Species—*Stachys affinis* Bunge (binomial name), and *Stachys sieboldii* Miq. and *Stachys tuberifera* Naudin. (synonyms). *S. affinis* is morphologically similar to *S. palustris* L., but it is shorter, thicker, and the tubers have a delicate, artichoke-like flavor [17,18].

*S. affinis* originates from central and northern China, where it has been cultivated since the 13th century [16]. Before its introduction to Europe, a related crop, *S. palustris*, was collected in nature and consumed as a vegetable. The Germanic peoples later used another relative, *S. recta*, as a medicinal plant [19]. The plant was cultivated in Europe from the 18th century onwards, with the first cultivation on a farm occurring in Crosne (Essonne Province), France in 1882. *S. affinis* is notable as the only labiate cultivated as a vegetable in Europe. It gained popularity in the early 20th century but was largely abandoned in the 1970s due to virus problems and the crop’s strong tendency to spread. In the late twentieth century, *S. affinis* gradually fell out of favor in Europe. However, following an international trend of rediscovery and revaluation, the Chinese artichoke has recently regained popularity. It is now available on the market and utilized in various culinary dishes [3,20,21].

Chinese artichokes grow up to 45 cm in height and tend to sprawl. They prefer well-drained soil in a sunny location and thrive best in ordinary garden soil that is not too heavy. Optimal growth is achieved in well-fertilized soil that remains moist throughout the growing season. These plants can endure waterlogged conditions in winter and are highly tolerant of high summer temperatures. In heavier soils, cleaning the tubers becomes more challenging, and in very heavy soil it may be beneficial to grow Chinese artichokes in containers. The tubers begin to sprout at temperatures above approximately 5 °C. It takes 5 to 7 months for the tubers to develop. The plant exhibits a runner growth habit, spreading indefinitely by rhizomes or stolons. The root pattern is rhizomatous, with underground stems sending out roots and shoots along their length. An average plant produces 20–30 tubers. Tubers should be planted between October and April, with the points facing up, at a depth of 4–8 cm. Tubers should be spaced 15–30 cm apart, in rows 45 cm apart. Alternatively, tubers can be left to sprout in trays or pots filled with damp compost and planted out when the leaves reach 8–10 cm in height. When the stems reach a height of 30 cm, they should be earthed-up to a depth of 8 cm. Foliage can be cut back if it becomes untidy, and flowers should be removed to concentrate the plant’s energy on tuber production. Harvesting should begin from October onwards, when the foliage starts to die down, and can continue over the winter months. Tubers can be left in the soil until needed, but the soil should be protected as it will make it easier to lift the tubers in frosty weather. Chinese artichokes can also be grown in pots, which is particularly recommended for areas with very heavy soil. They should be planted in a mixture of garden soil and good-quality compost, using one tuber per pot. Weeding is necessary, but care must be taken not to damage the root system. A sufficient water supply during the summer is crucial [20,21,22]. Harvesting occurs from November to March. It is important that the soil is not frozen during harvesting [23]. The storage of *S. affinis* tubers is challenging. Due to their thin skin, they can only be stored for a few days, or approximately a week in a refrigerator. 

Temperature, light, and water activity significantly impact the bioactivity and shelf life of Chinese artichoke (*S. affinis*). High temperatures can degrade sensitive bioactive compounds, such as antioxidants and polyphenols, reducing their health benefits. Light exposure, particularly UV light, can also diminish these compounds, although moderate UV exposure might stimulate certain beneficial phytochemicals. Controlled environments, like high tunnels, can help maintain a stable temperature and reduce light exposure. Water activity is crucial as high moisture levels can lead to microbial growth, decreasing shelf life. Proper storage in cool, dark, and low-humidity conditions can preserve the nutritional and bioactive properties of Chinese artichoke tubers, extending their shelf life [24].

## 3. Bioactive Compounds and Nutritional Value

The Chinese artichoke is rich in bioactive ingredients, according to available literature data, which are responsible for its health-promoting effects. These substances can be classified as primary and secondary metabolites. Primary metabolites are essential substances necessary for life, including nutrients, storage compounds, structural elements, and energy sources for plants. These include amino acids, proteins, fats, simple sugars, enzymes, nucleic acids, and chlorophyll. In contrast, secondary metabolites are organic substances that are not essential for plant growth but are metabolic products characteristic of fungi, bacteria, and higher plants. These include biominerals, vitamins, lipids, polyphenols, isoprenoids, and complex carbohydrates [2,25].

**Figure 1:**
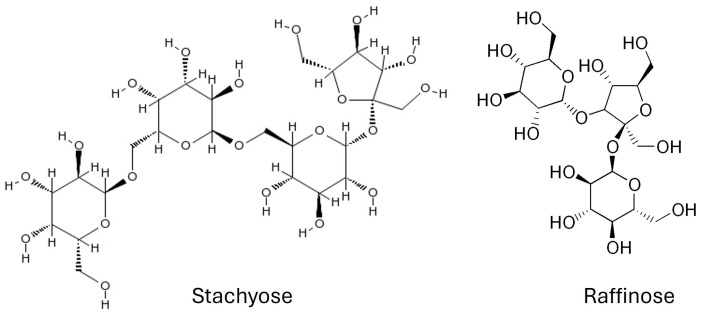
Chemical structures of stachyose and raffinose in tubers of *S. affinis*.

Simple sugars, organic acids, fats, and proteins significantly influence the taste, and nutritional and energy values of Chinese artichoke. According to Venditti et al. [3], 100 g of dried Chinese artichoke root provides 195 kcal. Carbohydrates, including simple sugars, are the dominant macronutrient group, comprising 36.94%, with simple sugars at 14.07%, proteins at 10.64%, and fats at 0.53%. Additionally, simple sugars are supplemented by stachyose, an oligosaccharide (tetrasaccharide) composed of galactose, fructose, and glucose, and amounting to 194.6 mg/g dry matter (dm), and raffinose at 42 mg/g dm [20], with stachyose purity at 87% (Figure 1). This oligosaccharide is not digested, but it is fermented in the large intestine by probiotic bacteria. Moreover, it acts as a prebiotic, serving as a substrate for probiotics, maintaining gut eubiosis, and inhibiting harmful bacteria proliferation. Due to its indigestibility, Chinese artichoke tubers are consumed in fermented form in China, making the oligosaccharides digestible [26]. However, stachyose can cause bloating and gastrointestinal issues in sensitive individuals. Despite these drawbacks, stachyose has hypoglycemic benefits by lowering blood glucose levels [27]. It is also used as a sucrose substitute, indicating the potential of Chinese artichoke tubers as a functional food ingredient. Another commonly occurring compound is succinic acid, a dicarboxylic acid. Beyond its role as a metabolite in the citric acid cycle, succinic acid exhibits health benefits, including anticonvulsant, anxiolytic, and antidepressant properties [3,28]. It also has hypoglycemic and cholesterol-lowering effects [29].

The Chinese artichoke is also rich in ash, comprising 8.44% dm, indicating a wealth of macro- and micro-elements. The tuber contains notably high potassium, phosphorus, calcium, magnesium, iron, and sulfur levels among the macroelements (Table 1). The most predominant microelements ranged from 0.15 (Nb) to 75.83 mg/kg (Na) (Table 2), and the presence of elements such as palladium, silver, cadmium, tin, antimony, lanthanum, tungsten, mercury, thallium, and uranium were below 0.10 mg/kg. The iron content is comparable to that found in Jerusalem artichoke [30]. Based on these dominant bioelements, the consumption of Chinese artichoke may offer significant health benefits. Another important component found in the tubers is dietary fiber, with a content of 35% dm. Dietary fiber is widely known to be a crucial part of the diet, positively affecting the digestive system, and acting as a prebiotic for probiotic bacteria in the gut microbiota, thus regulating bowel function [30]. Additionally, the use of Chinese artichoke in traditional Chinese medicine for treating heart diseases may be attributed to its high potassium content, as a diet rich in potassium can protect against cardiovascular diseases [31].

**Figure 2:**
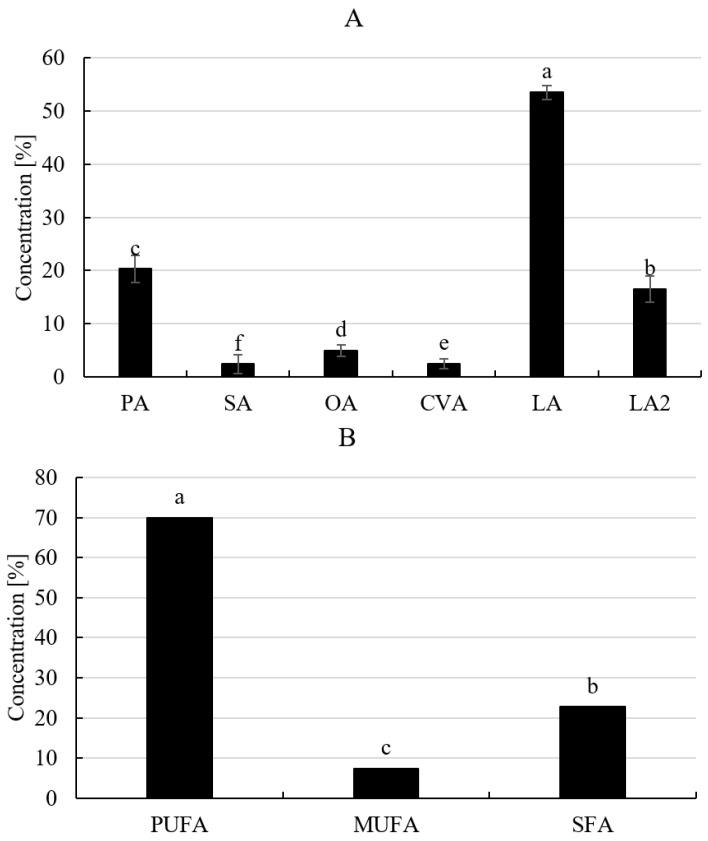
Profile of fatty acids (**A**) and the total percentage of fatty acid classes (**B**) in *S. affinis* tubers [3]; a–f; different letters (between morphological parts) within the same row indicate statistically significant differences (*p* < 0.05). Explanations: PA, palmitic acid (hexadecanoic acid); SA, stearic acid (Octadecanoic acid); OA, oleic acid ((*Z*)-9-octadecenoic acid); CVA, *cis*-vaccenic acid ((*Z*)-11-octadecenoic acid); LA, linoleic acid ((*Z*,*Z*)-9,12-octadecadienoic acid); LA2, linoleic acid ((*Z,Z*,*Z*)-9,12,15-octadecadienoic acid); PUFA, polyunsaturated fatty acids; MUFA, mono unsaturated fatty acids; SFA, saturated fatty acids [3].

**Figure 3:**
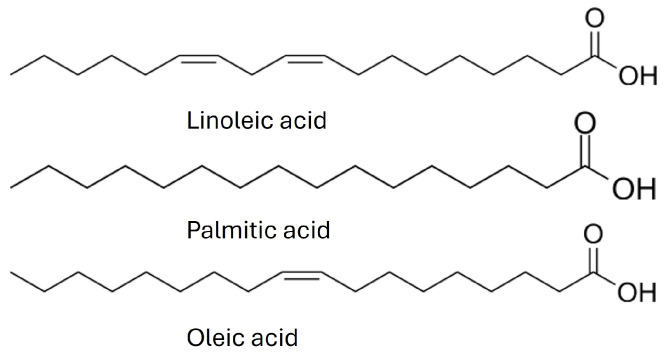
Chemical structures of the main fatty acids in the tubers of *S. affinis*.

*Stachys affinis* is characterized by an intriguing fatty acid profile. Among the identified fatty acids, linoleic acid was the predominant acid: linolenic > palmitic > oleic > *cis*-vaccenic > stearic (Figure 2 and Figure 3). This fatty acid profile differs from that of seeds from other *Stachys* L. species, where the dominant compounds were linoleic acid (27.1% to 64.3%), oleic acid (20.3% to 48.1%), and 6-octadecenoic acid (2.2% to 34.1%) [32]. This PUFA ratio is considered optimal for the prevention of certain diseases such as cardiovascular diseases, cancers, diabetes, obesity, rheumatoid arthritis, autoimmune diseases, depression, and asthma, with an ideal omega-6 to omega-3 ratio range of 1–4:1 [32,33]. Incorporating Chinese artichoke tubers into the diet is advisable based on the fatty acid content.

**Figure 4:**
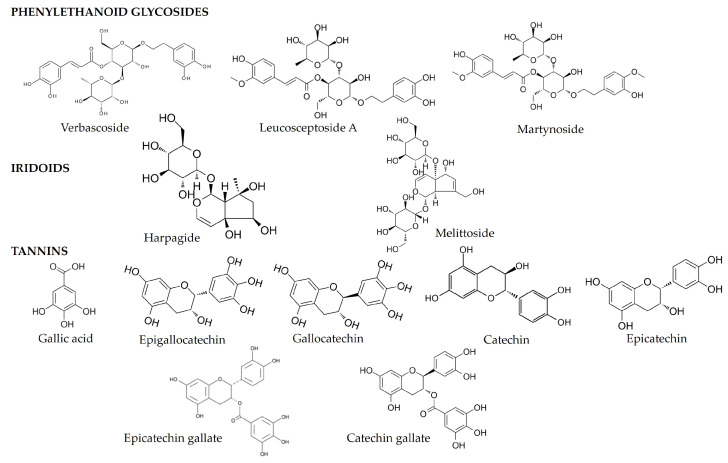
Chemical structures of the main polyphenolic compounds in tubers of *S. affinis*.

Chinese artichoke also contains polyphenolic compounds belonging to three classes: phenylethanoid glycosides (PhGs; such as verbascoside, leucosceptoside A, martynoside, and Stachysoside C), iridoids (such as harpagide, 8-*O*-acetyl-harpagide, melittoside, and 5-*O*-allosyloxyl-aucubin), and saponins (sieboldii saponin A)—compounds known for their bitter-sweet taste (Figure 4) [3,34]. These compounds contribute to the tubers flavor profile. Verbascoside is commonly found in *Stachys* L. species such as *S. beckeana*, *S. anisochila*, *S. alpina* subsp. *dinarica*, *S. plumosa*, *S. tymphaea*, and *S. germanica* subsp. *Salviifolia* [3,35,36,37]. It is also widespread in related botanical families within the Lamiales order, such as Orobanchaceae, Plantaginaceae, and Scrophulariaceae [38,39,40,41]. Verbascoside is metabolically significant in the genus Asteridea and co-occurs with iridoid glycosides. This compound is valuable for its anti-inflammatory, anticancer, antioxidant, and antihistamine properties [38,39,40,41]. Other PhGs present in the tubers also play a significant role as antioxidants and exhibit estrogenic properties [42]. Iridoid glycosides like harpagide, 8-*O*-acetyl-harpagide, 5-*O*-allosyloxyl-aucubin, and melittoside, commonly found in most *Stachys* L. species, act as chemotaxonomic markers and have health benefits including antioxidant, anticancer, antiviral, anti-inflammatory, analgesic, and anti-osteoporosis effects [28].

**Figure 5:**
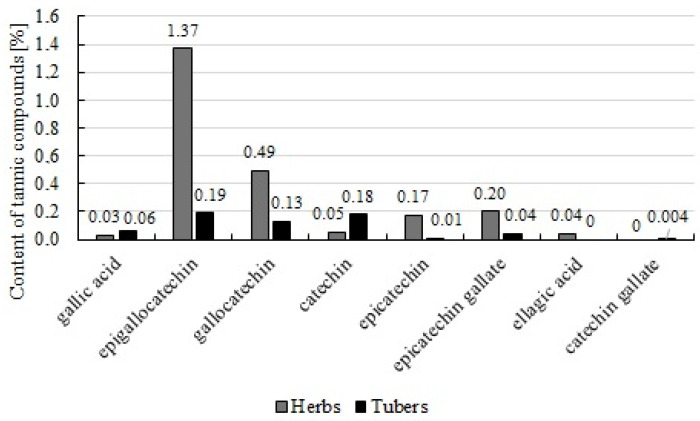
The content of tannin compounds in herbs and tubers of *S. sieboldii* [46].

Studies by Guo et al. [43] indicate that the phenolic content in Chinese artichoke tubers is 587.33 mg TAE/g dm, and flavonoids are 60 mg QE/g dm. These flavonoid values are similar to those obtained by Lee et al. [44]. Kang et al. [45] noted polyphenols content ranging from 7.18–37.25 mg/g, and flavonoids from 0.21–5.21 mg/g. In Lee et al. [46], polyphenols were 124.61 mg GAE/g and flavonoids were 45.2 mg QE/g. These results indicate the presence of flavonoids, though detailed identification is currently lacking. Both tubers and herbs contain tannic substances, including gallic acid, epigallocatechin, gallocatechin, catechin, epicatechin, epicatechin gallate, ellagic acid (only in herbs), and catechin gallate (only in tubers) (Figure 4 and Figure 5) [47]. Considering the above, Chinese artichoke tubers represent an underappreciated dietary component and could act as a functional food ingredient or sugar substitute.

## 4. Health-Promoting Properties

For thousands of years, herbs have been used in the prevention and treatment of various diseases in both humans and animals [47]. In recent years, there has been a growing interest in plants from the Lamiaceae family due to their valuable chemical composition, which contributes to their unique health benefits. Various species of the L. family have been extensively analyzed to confirm their beneficial health effects [3,4,9,38,43]. However, these studies have predominantly focused on the aerial parts of the plants. In contrast, *S. affinis* Bunge (synonym: *S. sieboldii* Miq.) has not only edible aerial parts, but also edible roots with a unique composition. This has led to a focus on a detailed understanding of the chemical compounds of this plant.

In traditional medicine, not only the leaves, stems, and flowers are used, but also the tubers, as in the case of *S. affinis* Bunge, or rhizomes in other *Stachys* L. plants. They have been used against many infections, including urinary tract infections, heart diseases, colds, tuberculosis, etc., as well as in treating dementia and various gastrointestinal problems [31,48,49,50], demonstrating their anti-toxic, anti-inflammatory, and antibacterial effects [31,51]. 

### 4.1. Antioxidant and Antimicrobial Activity

In the study by Venditti et al. [3], the effects of *Stachys affinis* Bunge tuber extracts were evaluated on cancer cell lines such as neuroblastoma (SHSY-5Y), colon adenocarcinoma (Caco-2), and chronic myeloid leukemia (K562) to combat the oxidative damage induced by tetra-butyl hydroperoxide (*t*-BHP). Intracellular reactive oxygen species (ROS) levels were analyzed in these cells. K562 cells were treated with 100 µL *t*-BHP, while the other cells received 200 µL *t*-BHP, and all cells received increasing concentrations of an ethanol extract from Chinese artichoke tubers, ranging from 0.03 to 0.5 mg/mL. It was found that the ethanol extracts did not modify the intracellular ROS levels in vitro but effectively inhibited their formation in a dose-dependent manner in all tested cells. The highest concentration of 0.5 mg/mL completely inhibited ROS formation in all tested cells. Significant ROS inhibition was observed even at the lowest dose of 0.03 mg/mL, reducing ROS by 44% in Caco-2 cells (EC_50_ = 0.026 mg/mL), 23% in SH-SY5Y cells (EC_50_ = 0.05 mg/mL), and 82% in K562 cells (EC_50_ = 0.0023 mg/mL) [3]. This significant inhibition of excessive ROS formation and protection against oxidative stress (OS) is mainly attributed to the PhGs [3].

Additionally, the antioxidant activity of individual polyphenolic compounds identified in *S. affinis* Bunge tubers was assessed. Melittoside and 5-allosyloxyl-aucubin exhibited inhibitory effects against DPPH and ABTS radicals [3,4,9]. Guo et al. [43] evaluated antiradical properties using ABTS and DPPH assays, superoxide-scavenging activity, nitric-oxide-scavenging activity, and metal-chelating activity of five fractions of ethanol extract from the tubers, confirming that all fractions had high activity in protecting against excessive ROS formation. The ethyl acetate fraction showed the highest DPPH activity (IC_50_ = 0.85 µg/mL). These studies confirmed the exceptional antioxidant properties of *S. affinis* Bunge tubers and their potential use as natural antioxidants [43].

In a separate study conducted by Lee et al. [49], the effects of tuber extracts on H_2_O_2_-induced ROS production in HT-1080 cell lines were evaluated. The n-hexane fraction effectively inhibited DNA damage caused by hydrogen peroxide and oxidative stress, and increased glutathione production [49]. Acetone and methylene chloride extracts of *S. sieboldii* Miq. showed higher inhibition of ROS production compared to methanol extracts, with 0.25 mg/mL of the acetone/methylene chloride extracts inhibiting ROS production by 60%. This effect is mainly attributed to the presence of polyphenolic compounds in the plant [49]. In studies conducted by Lee et al. [46], 400 µg/mL of the tuber extract showed 53% protection against OS in HepG2 cell lines.

**Figure 6:**
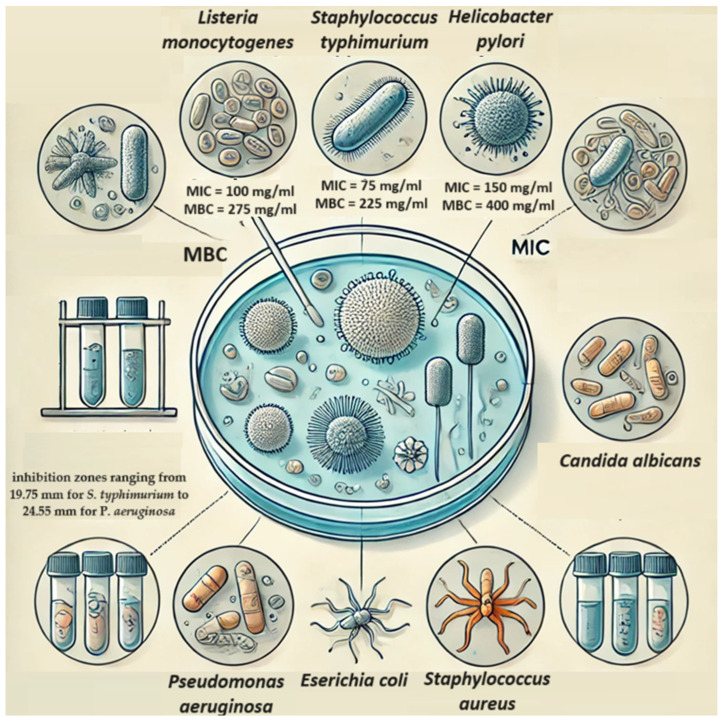
The antibacterial activity of Chinese artichoke tuber extracts.

Studies evaluating the antibacterial activity of Chinese artichoke tuber extracts noted inhibitory effects against *Listeria monocytogenes*, *Staphylococcus typhimurium*, and *Helicobacter pylori*. The minimum inhibitory content (MIC) for *L. monocytogenes*, *S. typhimurium*, and *H. pylori* was 100, 75, and 150 mg/mL of extract, respectively (Figure 6). The minimum bactericidal content (MBC) was 275, 225, and 400 mg/mL, respectively [46]. Additionally, studies conducted by Slobodianiuk et al. [51] indicate antibacterial and antifungal properties of Chinese artichoke tuber extracts against *S. typhimurium*, *Pseudomonas aeruginosa*, *Escherichia coli*, *Staphylococcus aureus*, and *Candida albicans*, with inhibition zones ranging from 19.75 mm for *S. typhimurium* to 24.55 mm for *P. aeruginosa* [51].

### 4.2. Anti-Obesity, Anti-Dyslipidemic and Anti-Hypoglycemic Effects

In the research carried out by Lee et al. [52], the therapeutic effects of *S. sieboldii* (= *S. affinis*) tuber extract on dyslipidemia and obesity were evaluated in an in vivo animal model (rats) induced by a high-cholesterol and high-fat (HFC) diet. The study also investigated the lipid mechanism in the context of the tuber extracts’ impact. It was noted that the HFC diet led to an increase in body weight and the food efficiency ratio (FER) in rats. However, supplementation with Chinese artichoke tuber extract in the HFC diet resulted in a dose-dependent decrease in body weight gain and FER levels. Additionally, HFC-fed rats showed an increase in liver weight, mesenteric adipose tissue (MAT), white adipose tissue (WAT), perirenal adipose tissue (PAT), and retroperitoneal adipose tissue (RAT), whereas supplementation with the extract reduced these parameters, with the most significant change observed in MAT. The HFC diet did not affect the EAT index, but the addition of the extract to the diet reduced this parameter dose-dependently. Supplementation with the extract also reduced the expression of adipogenic genes, markers of liver cell necrosis [53], and fasting glucose levels. Moreover, supplementation with the Chinese artichoke extract in the HFC diet improved lipid profiles, reduced lipid accumulation in the liver, serum, and adipose tissue, decreased CVD risk parameters, enhanced fat metabolism at the transcriptional level, and increased fecal fat excretion. The most beneficial results in counteracting obesity and dyslipidemia were observed with a diet containing 5% *S. sieboldii* tuber extract. Therefore, the findings suggest that incorporating Chinese artichoke tuber extracts into the daily diet could improve obesity symptoms [52].

Furthermore, studies by Slobodianiuk et al. [51] evaluated the hypoglycemic effect of *S. sieboldii* tuber extract in an in vivo model. The insulin resistance model induced by dexamethasone was created by subcutaneously administering 4 mg/kg of dexamethasone for 4 days to rats weighing 180–230 g. Effective hypoglycemic action was noted in rats administered tuber extract at doses of 25 and 50 mg/kg. It was also noted that with increasing doses, the activity progressively decreased. The most pronounced hypoglycemic effect was observed at a dose of 25 mg/kg, while at 50 mg/kg the effect was comparable to that of the officially approved herbal mixture “Arfazetin”. These results indicate the potential hypoglycemic action of the extracts, expanding the tubers’ application spectrum and making them more attractive in the food market [51].

### 4.3. Anticholinesterase, Neuroprotective, Antiproliferative, Anti-COPD Effects

In the scientific study by Ravichandran et al. [50], the molecular mechanism of the neuroprotective effect of *S. sieboldii* tuber extract was evaluated, focusing on its ability to enhance learning and memory. The study was conducted in an in vivo animal model of scopolamine (SCOP)-induced amnesia [54]. Particular attention was given to cholinergic neurotransmission. The results indicated that the Chinese artichoke tuber extracts improved cognitive functions and alleviated SCOP-induced memory impairments by regulating the NGF-BDNF-CREB signaling pathway. Additionally, the extract increased the concentrations of acetylcholine (ACN) and acetylcholine esterase (CAT) and inhibited the activity of acetylcholinesterase (AChE) in the hippocampus (the memory center) and the frontal cortex, compared to the SCOP group. This confirmed the protective effect of the extract against memory impairment. The study also suggested that the extract could prevent memory loss by inhibiting neuronal cell death programming and/or activating neurotrophic factors. Moreover, the tuber extract showed the ability to activate synaptic and extra-synaptic GABAA receptors, playing a crucial role in neuronal growth and neuroprotection through the activation and regulation of these receptors [50]. It was suggested that a daily intake of about 2430 mg of tuber extract, providing 0.83 mg/day of choline for a 60 kg person, as a dietary supplement could improve memory and prevent dementia [50]. These conclusions were also confirmed by other authors [54]. However, further clinical studies on humans are necessary to unequivocally confirm these effects.

**Figure 7:**
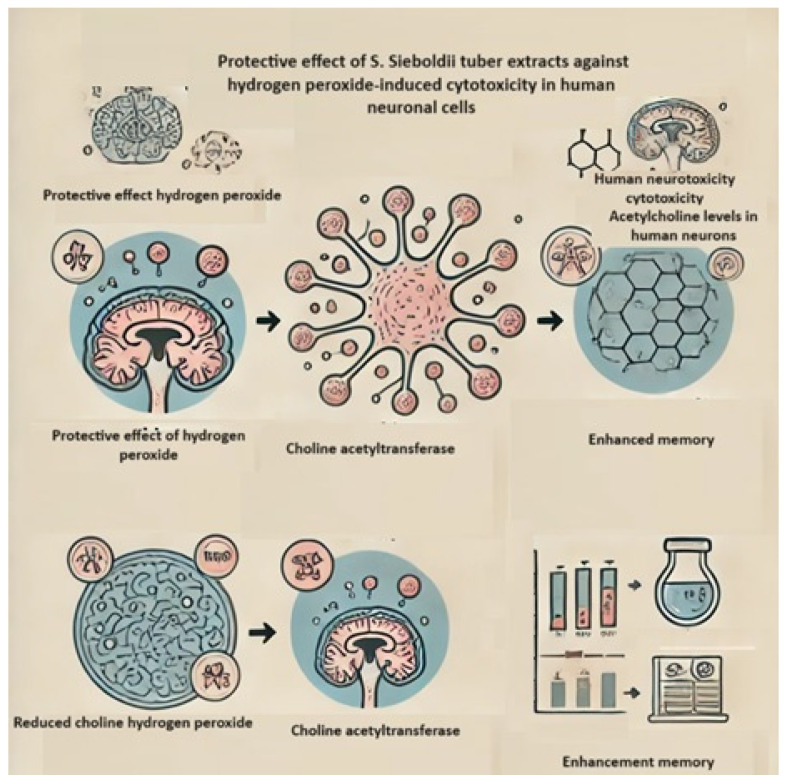
The protective effect of *S. sieboldii* tuber extracts against hydrogen peroxide-induced cytotoxicity in human neuroblastoma cells (SK-N-SH) and memory enhancement in mice.

The protective effect of *S. sieboldii* tuber extracts against hydrogen peroxide-induced cytotoxicity in human neuroblastoma cells (SK-N-SH) and memory enhancement in mice was also evaluated (Figure 7) [55]. It was shown that the Chinese artichoke tuber extract partially antagonized the effect of hydrogen peroxide (150 µL) on cell proliferation (in vitro model). Additionally, in an in vivo mouse model, a 500 mg/kg dose of tuber extract significantly increased transition delay times and enhanced memory. Memory enhancement was also observed in amnesia models, where the extract increased fear memory by elevating ACN and choline acetyltransferase levels in both the hippocampus and the cerebral cortex [55,56]. This effect was further confirmed by Kim et al. [57], who evaluated the neuroprotective effects of bread containing *Aster scaber* Thunb. and *S. sieboldii* Miq. against ethanol- or H_2_O_2_-induced neuronal cell death. The authors also assessed the effect of bread enriched with *S. sieboldii* extract against hydrogen peroxide- or ethanol-induced cytotoxicity in SK-N-SH cells, noting a reduction in intracellular ROS levels and a limiting of ethanol and hydrogen peroxide cytotoxicity in the analyzed cells.

Chinese artichoke tuber granules were also tested for chronic obstructive pulmonary disease (COPD) in an in vivo clinical study [58]. The patient group included 120 individuals suffering from stable COPD. Over 12 weeks, significant differences in the concentrations of pro-inflammatory factors IL-17A and TH17, and anti-inflammatory factors IL-10 and IFN-γ in plasma were noted. The pro-inflammatory factors were lower, and the anti-inflammatory factors were higher than in the control group. The results from the Saint George Respiratory Questionnaire (SGRQ) showed significantly higher scores in the group taking the Chinese artichoke extract. After 48 weeks of observation, the frequency of adverse effects in patients taking the artichoke granules decreased by 47.9%. Thus, the Chinese artichoke granules reduced pro-inflammatory factors and increased anti-inflammatory factors in COPD patients, mitigating adverse effects [58].

### 4.4. Anti-Inflammatory, Anticancer, and Antinephritic Effects

In a study by Slobodianiuk et al. [59], the anti-inflammatory effect of *S. sieboldii* herb extract was analyzed in an in vivo model of carrageenan-induced paw edema in rats. The study involved 30 rats weighing between 180 and 220 g, and paw volume was measured using a mechanical oncometer. It was noted that supplementation with the herb extract at doses ranging from 5 to 25 mg/kg reduced edema in the tested animals, with the most significant anti-inflammatory effects observed at a dose of 10 mg/kg. The effectiveness of the herb extract was demonstrated in the initial hours of inflammation dynamics, influencing serotonin, histamine, and leukotrienes as mediators of the acute inflammatory phase. This suggests the potential of this plant in anti-inflammatory treatment, though further clinical studies are needed to confirm its efficacy [59]. In another study on the anti-inflammatory effects of *S. sieboldii* tuber extract [46], it was shown that the extracts effectively inhibited the secretion of inflammatory cytokines TNF-α, IL-6, and IL-1β in a dose-dependent manner.

**Figure 8:**
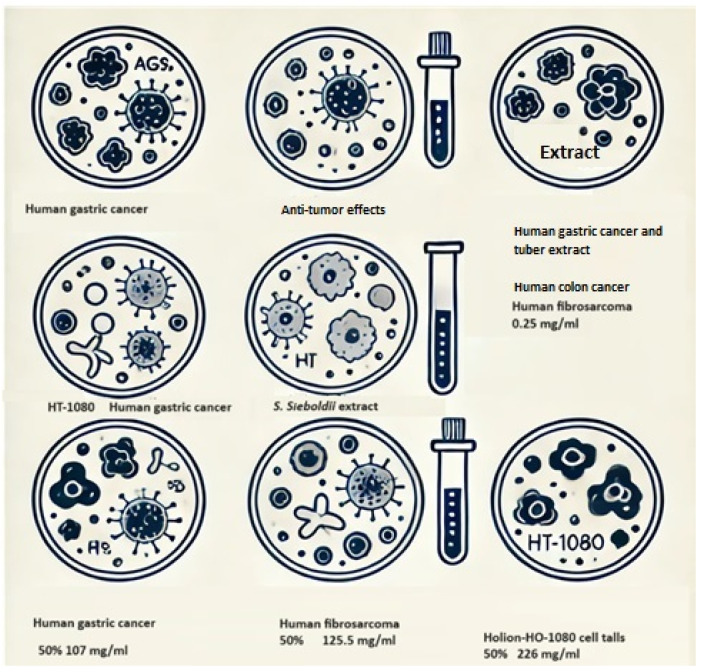
The antitumor effects of *S. sieboldii* tuber extracts on human gastric cancer (AGS), human fibrosarcoma (HT-1080), and colon cancer (HT-29) cell lines, using icons and diagrams to represent the biological processes and effects without depicting animals or humans.

The antitumor effects of tuber extracts were evaluated against human gastric cancer (AGS), human fibrosarcoma (HT-1080), and colon cancer (HT-29) cell lines [60]. The n-hexane and 85% MeOH extracts from *S. sieboldii* MIQ. inhibited AGS cell growth by 50% at a concentration of 0.1 mg/mL and inhibited the proliferation of HT-29 and HT-1080 cells by 50% and 60% at a concentration of 0.25 mg/mL, respectively. These high antiproliferative activities are attributed to the bioactive compounds present in the plant (Figure 8) [60].

Acteoside (ACT) was extracted from *S. sieboldii* tubers and its effect on anti-GBM crescentic nephritis was evaluated in an animal model (rats). First, anti-GBM serum was administered to induce the disease, followed by ACT treatment. ACT inhibited the increase in proteinuria and reduced the production of anti-rabbit γ-globulin antibodies in the plasma, as well as decreased creatinine and cholesterol levels. Additionally, ACT inhibited hypercellularity, crescent formation, fibrinoid necrosis of glomeruli, and capillary wall adhesion to Bowman’s capsule, indicating its potential as an antinephritic agent [35].

### 4.5. Microbiota Modulating Effects

**Figure 9:**
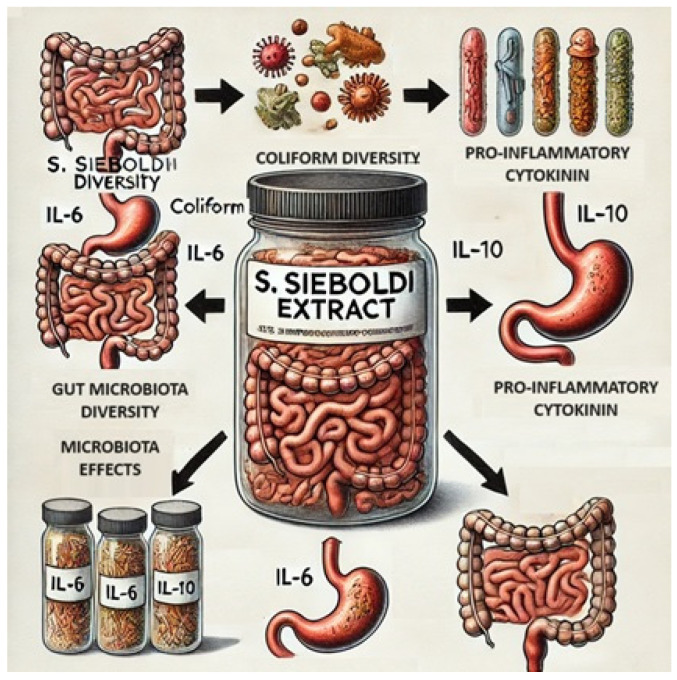
Microbiota modulating effects of *S. affinis* tuber extract.

A study by Na et al. [61] evaluated the effect of *S. sieboldii* tuber extract supplementation on gut microbiota composition and diversity, as well as cytokine production in mice. The results indicated that mice supplemented with the extracts had lower levels of coliform and aerobic bacteria in their feces compared to the control groups. An increase in beneficial gut bacteria such as *Ruminococcaceae* and *Akkermansia muciniphila*, and a significant decrease in harmful bacteria such as *Enterobacteriaceae*, including *Escherichia coli* and *Bacteroides* sp., were observed. Additionally, there was significantly lower mRNA expression of pro-inflammatory cytokine IL-6 and anti-inflammatory cytokine IL-10 in the lymph nodes of supplemented mice. This is likely related to the higher concentration of beneficial microorganisms and lower content of harmful microorganisms in the gut microbiota (Figure 9) [61].

## 5. Applications in Food Manufacturing

The tubers possess a crunchy texture and a sweet, nutty flavor. They can be consumed raw, pickled, dried, or cooked. This vegetable’s versatility leads to its incorporation into various dishes across many countries’ cuisines. Preparation methods are similar to those used for Jerusalem artichokes. The leaves can be dried and made into tea [26].

In Chinese and Japanese cuisines, *S. affinis* is primarily pickled. Specifically, it is the tuber is part of Osechi, a dish prepared to celebrate the Japanese New Year [62]. After being pickled and dyed red by leaves of Perilla (red shiso), it is referred to as chorogi. In Korea, it is known as choseokjam. In French cuisine, the cooked tuber is often served with dishes referred to as japonaise, or Japanese-styled [63]. Once reduced to powder, they are used to prepare rice cookies and added to bread, known as ‘mayday flour’ [64,65].

**Figure 10:**
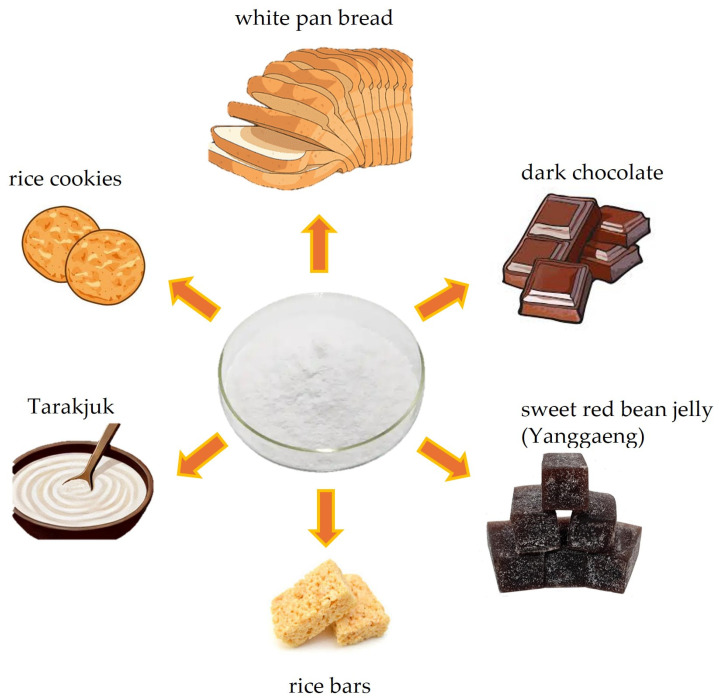
Applications of *S. affinis* tubers in food manufacturing.

Studies have also evaluated the potential use of Chinese artichoke tubers as a functional food ingredient by assessing the quality of selected food products such as rice nutritional bars [66], white pan bread [67], Tarakjuk [68], chocolate [69], Yanggaeng [70], and cookies [71] (Figure 10).

Joo et al. [66] assessed the quality of rice nutritional bars enriched with Chinese artichoke tuber powder. It was reported that the amount of polyphenols and the antiradical activity increased with the addition of the powder. Additionally, elasticity and cohesiveness increased, while firmness, gumminess, and chewiness decreased with higher powder content. The most favorable nutritional properties and health benefits were observed in products substituting 15% of rice flour with Chinese artichoke powder [66].

Jeon et al. [67] evaluated the quality of white pan bread with Chinese artichoke powder as a functional additive. Increasing the powder content reduced the bread’s volume and specific volume while increasing its weight, firmness, gumminess, chewiness, and stickiness, and reducing cohesiveness and elasticity. The optimal addition of *S. affinis* powder was determined to be 9% [67].

Tae et al. [68] examined the physicochemical and sensory characteristics of Tarakjuk with Chinese artichoke tuber powder. They found that increasing the powder content increased DPPH radical-scavenging activity, spreadability, and moisture content while decreasing viscosity and acidity. A 10% addition was deemed optimal for sensory and nutritional properties [68].

The effect of adding 10–20% Chinese artichoke powder on the nutritional value of chocolate was also assessed [69]. The results showed an increase in ash, protein, lipids, polyphenols, flavonoids, and antioxidant activity, while hardness, cohesiveness, elasticity, adhesiveness, gumminess, and chewiness decreased.

Choi et al. [70] evaluated the quality and nutritional value of Yanggaeng with Chinese artichoke powder. The study indicated a decrease in total extract content, pH, chewiness, and cohesiveness, while polyphenol content, antioxidant activity, and moisture increased with higher powder content. A 4% addition was optimal for improving the product’s quality and nutritional value.

Na et al. [71] assessed the antioxidant capacities of *S. sieboldii* MIQ and ginseng powders and their effects on the quality characteristics of cookies. The results indicated that the powder positively influenced the nutritional value, health benefit, and sensory properties of the final product.

## 6. Conclusions

*Stachys affinis* Bunge, known as Chinese artichoke, is a perennial plant from China, attracting significant scientific interest for its rich content of bioactive compounds, including antioxidants, polyphenols, and prebiotics. The tubers are also high in essential nutrients and dietary fiber, making them valuable in the diet and as a functional food ingredient. Studies have shown that incorporating Chinese artichoke tuber powder into products like rice bars, bread, and chocolate enhances their nutritional and sensory properties. These products exhibit increased polyphenol levels, improved texture, and enhanced nutritional profiles. Chinese artichoke also displays significant anti-inflammatory, neuroprotective, and antibacterial activities. It inhibits inflammatory cytokines such as TNF-α, IL-6, and IL-1β, improves cognitive functions by regulating the NGF-BDNF-CREB signaling pathway, and shows antiproliferative effects on cancer cell lines like AGS, HT-1080, and HT-29. Botanically, *S. affinis* belongs to the Lamiaceae family and grows up to 45 cm in height. The plant thrives in well-drained soil and sunny locations. Additionally, Chinese artichoke modulates gut microbiota, increasing beneficial bacteria and reducing harmful ones, which is associated with lower pro-inflammatory cytokines. Overall, Chinese artichoke is a versatile plant with numerous health benefits, making it a promising candidate for further research and use in the food industry.

## Figures and Tables

**Table 1:** The content of major mineral elements [3].

Major Mineral Elements	Concentration [g/kg]
Magnesium (Mg)	2.22 ± 0.11
Phosphorus (P)	4.10 ± 0.35
Sulfur (S)	1.07 ± 0.10
Potassium (K)	23.61 ± 2.30
Calcium (Ca)	3.83 ± 0.37
Iron (Fe)	1.38 ± 0.08

**Table 2:** The content of minor mineral elements [3].

Minor Mineral Elements	Concentration [mg/kg]
Lithium (Li)	1.94 ± 0.12
Boron (B)	12.25 ± 1.23
Sodium (Na)	75.83 ± 13.91
Titanium (Ti)	53.42 ± 4.13
Vanadium (V)	3.91 ± 0.15
Chromium (Cr)	3.28 ± 0.18
Manganese (Mn)	26.07 ± 3.74
Cobalt (Co)	0.44 ± 0.05
Nickel (Ni)	2.14 ± 0.17
Copper (Cu)	17.27 ± 1.64
Zink (Zn)	23.60 ± 1.33
Gallium (Ga)	3.76 ± 0.23
Germanium (Ge)	0.22 ± 0.01
Arsenic (As)	0.62 ± 0.04
Selenium (Se)	0.82 ± 0.05
Rubidium (Rb)	10.68 ± 0.65
Strontium (Sr)	15.10 ± 1.33
Zirconium (Zr)	0.45 ± 0.15
Niobium (Nb)	0.15 ± 0.04
Molybdenum (Mo)	0.59 ± 0.03
Cesium (Cs)	0.25 ± 0.01
Barium (Ba)	12.16 ± 0.87
Lead (Pb)	1.49 ± 0.10
